# The structure of the cognitive reserve in Alzheimer’s disease

**DOI:** 10.1192/j.eurpsy.2021.1911

**Published:** 2021-08-13

**Authors:** A. Sidenkova

**Affiliations:** Psychiatry, Ural State Medical University, Yekaterinburg, Russian Federation

**Keywords:** Alzheimer’s disease, cognitive reserve, Pathological brain aging, cognitive functions during aging

## Abstract

**Introduction:**

Alzheimer’s disease is common among the modern population. Emotional support for caregivers and a stable social role contribute to the maintenance of cognitive resources in Alzheimer’s.

**Objectives:**

To investigate the protective mechanisms that protect the brain from premature aging

**Methods:**

Clinical, sociological

**Results:**

Violations of the higher cerebral functions of speech, gnosis, praxis are the neuropsychological basis for the development of psychological symptoms of dementia. Speech disorders, gnosis disorders contribute to the formation of painful ideas, perception disorders, eating disorders and affective symptoms. The level of functional activity is low and does not depend on the state of severe microsocial dementia, total aphasia and apraxia. The social, work, family and marital status of caregivers for patients with moderate to severe dementia is declining. An increase in the degree of dementia can reduce the functional activity of the caregiver. Caregivers often suffer from neurotic, affective and other mental disorders. A person with dementia plays a subordinate role in the family. Changing roles in the family occurs when the patient has delusion, agitation / aggression, anxiety, unstable mood / irritability. In these cases, the interpersonal distance in the “care-patient” pair increases. Caregivers have a high level of emotional involvement in the care process. A change in the role of the family, a change in place of residence, and a high level of “expressive” emotions of the guardian negatively affect the formation of psychosis, anxiety and aberrant behavior in patients with dementia. Microsocial factors influence cognitive retention in dementia

**Conclusions:**

Protective psychosocial factors strengthen the cognitive reserve

**Disclosure:**

No significant relationships.

